# Propofol versus remifentanil sedation for elective gastroscopy: a randomized controlled trial

**DOI:** 10.1590/1806-9282.20260891

**Published:** 2026-10-23

**Authors:** Cansu Ofluoğlu, Doğa Meriç Yükselen, Arsen Güngör Ay, Ayşe Günler Gayretli, Öznur Demiroluk, Arzu Yıldırım Ar

**Affiliations:** 1 University of Health Sciences, Fatih Sultan Mehmet Training and Research Hospital, Department of Anesthesiology and Reanimation – Istanbul, Turkiye.

**Keywords:** Conscious sedation, Gastroscopy, Patient safety, Propofol, Postoperative period, Remifentanil

## Abstract

**OBJECTIVE:**

Propofol and remifentanil are widely used for elective gastroscopy sedation. The aim of this randomized controlled trial was to compare their safety profiles, patient-reported recovery quality, and endoscopist satisfaction.

**METHODS:**

This single-center randomized controlled trial enrolled 88 adults (American Society of Anesthesiologists I–III) undergoing elective gastroscopy, allocated 1:1 to propofol (Group P, n=44) or remifentanil (Group R, n=44). Sedation depth was standardized using the Bispectral Index (target 60–80) and Ramsay Sedation Scale (target 2–3). The primary outcome was the Quality of Recovery-15 score at 30 min post-procedure; secondary outcomes included complication incidence, awakening time, and endoscopist satisfaction.

**RESULTS:**

Quality of Recovery-15 scores were comparable (median 140 vs. 140; median difference 0, 95%CI -1.0 to 1.0; p=0.95). Total complication incidence was 0% in Group P versus 61.4% in Group R (p<0.001), comprising bradycardia (27.3%), postoperative nausea and vomiting (20.5%), and desaturation (13.6%). Awakening time was shorter in Group R (3 vs. 60 s; p<0.001). Endoscopist satisfaction was significantly higher in Group P (90.9 vs. 56.8% excellent scores; p=0.003).

**CONCLUSION:**

Despite comparable patient-reported recovery quality, propofol was associated with substantially fewer perioperative adverse events (0 vs. 61.4%) and higher endoscopist satisfaction, supporting its preferential use in routine elective gastroscopy sedation.

**Clinical Trial Registration Number:**

ClinicalTrials.gov, NCT07336628. Registered January 2, 2026.

## INTRODUCTION

Gastroscopy is among the most frequently performed endoscopic procedures worldwide, and its use continues to increase^1^. Sedation is routinely employed to reduce anxiety, suppress the gag reflex, and improve procedural tolerability^2,3^.

Propofol, a short-acting intravenous hypnotic, offers rapid onset and antiemetic properties. Remifentanil, an ultra-short-acting μ-opioid agonist with a context-sensitive half-time of approximately 3–4 min, is attractive for brief day-case procedures^4,5^. Each agent carries distinct adverse effects: propofol causes dose-dependent cardiorespiratory depression, while remifentanil may cause bradycardia, respiratory depression, and postoperative nausea and vomiting (PONV)^4,5^.

Existing comparative studies have predominantly focused on colonoscopy or endoscopic retrograde cholangiopancreatography (ERCP), with gastroscopy-specific data limited and largely restricted to single safety endpoints^6,7^. The Quality of Recovery-15 (QoR-15) is a validated 15-item scale assessing five recovery dimensions; its application as a primary endpoint in gastroscopy sedation has not been previously reported^8,9^.

This trial compared propofol and remifentanil sedation for elective diagnostic gastroscopy, using QoR-15 as the primary outcome. We hypothesized that propofol would demonstrate a more favorable safety profile with comparable patient-reported recovery quality.

## METHODS

### Study design and ethical approval

This prospective, single-center, randomized controlled trial was conducted between February and April 2026 at the Departments of Anesthesiology and Gastroenterology, University of Health Sciences, Fatih Sultan Mehmet Training and Research Hospital, Istanbul, Turkiye. Ethics committee approval was obtained (Code: 2025-KAEK-43; Decision No: 2025/20/1260; Date: November 4, 2025). Written informed consent was obtained from all participants. The study was conducted in accordance with the Declaration of Helsinki and prospectively registered at ClinicalTrials.gov (NCT07336628). This manuscript was prepared in accordance with Consolidated Standards of Reporting Trials (CONSORT) 2010 guidelines.

### Study population

Adult patients (age ≥18 years, American Society of Anesthesiologists [ASA] I–III) scheduled for elective diagnostic gastroscopy under sedation were enrolled. Exclusion criteria were: patient refusal, emergency procedures, known allergy to propofol, remifentanil, or egg/soy products, and ASA IV or above.

### Randomization and blinding

Eligible patients were randomized in a 1:1 ratio using a computer-generated block randomization sequence with block sizes of 4 and 8. The allocation sequence was prepared by an investigator who was not involved in patient recruitment, sedation, or outcome assessment and placed into sequentially numbered, sealed, opaque envelopes. Immediately before sedation, the attending anesthesiologist opened the next envelope in sequence to determine treatment allocation. Patients and the investigator assessing the QoR-15 questionnaire were blinded to treatment allocation. Complete endoscopist blinding was not feasible because of the distinct visual appearances of the study drugs.

### Sedation protocols

Standard monitoring included electrocardiography, non-invasive blood pressure, pulse oximetry, and Bispectral Index (BIS) monitoring (BIS Vista, Medtronic). Supplemental oxygen was administered at 6 L/min via nasal cannula throughout the procedure. Group P received propofol (Propofol 1%, Fresenius Kabi) at an initial dose of 0.5–1 mg/kg, with additional doses administered as required to maintain a BIS value of 60–80 and a Ramsay Sedation Scale (RSS) score of 2–3. Group R received remifentanil (Ultiva, GlaxoSmithKline) as a 1 μg/kg intravenous loading dose followed by a continuous infusion of 0.025–0.1 μg/kg/min. The infusion rate was titrated throughout the procedure to maintain the same target BIS (60–80) and RSS (2–3) values and was discontinued immediately after completion of the gastroscopy. No midazolam premedication was administered.

### Outcome measures

The primary outcome was the QoR-15 score measured 30 min after the procedure. The QoR-15 is a validated 15-item patient-reported measure of postoperative recovery^10^, and quality of recovery is a recommended core endpoint for patient comfort in perioperative trials^11^. The validated Turkish version of the questionnaire was administered^12^. Because all participants underwent ambulatory day-case gastroscopy and were discharged on the same day, the questionnaire was administered before discharge to evaluate early postoperative recovery. Secondary outcomes included awakening time, perioperative complication incidence, procedure duration, time to procedure initiation, time to discharge, and endoscopist satisfaction assessed using a five-point Likert scale. Awakening time was defined as the interval, in seconds, from completion of the gastroscopy to the patient’s first appropriate response to a verbal command. Complications were defined as hypotension (mean arterial pressure (MAP) <65 mmHg or >20% decrease from baseline), bradycardia (heart rate [HR] <50 bpm), desaturation (SpO^2^ <90% persisting for more than 10 s), and PONV.

### Statistical analysis

Sample size was calculated based on a QoR-15 minimal clinically important difference (MCID) of 8.0 points and standard deviation (SD) of 12 points (Cohen’s d=0.67), yielding 37 patients per group (80% power, two-sided alpha 0.05); with a 20% dropout allowance, 44 per group were enrolled. Continuous variables are presented as median [interquartile range (IQR)] and compared using the Mann-Whitney U test; categorical variables were compared using chi-square or Fisher’s exact test. Risk differences and risk ratios with 95%CI were calculated for binary outcomes. Analyses were performed using IBM SPSS v.25. A two-sided p<0.05 was considered significant.

## RESULTS

### Participant flow and baseline characteristics

Of 126 patients assessed, 38 were excluded (22 ASA IV or above, 6 emergency procedures, 8 declined consent, 2 other reasons). The remaining 88 were randomized; 44 to Group P and 44 to Group R. No dropouts occurred; all were included in the intention-to-treat analysis (Figure 1). Baseline characteristics were comparable between groups (Table 1). The median total cumulative propofol dose was 160 (150–170) mg, whereas the median total cumulative remifentanil dose was 200 (200–280) μg.

**Figure 1:**
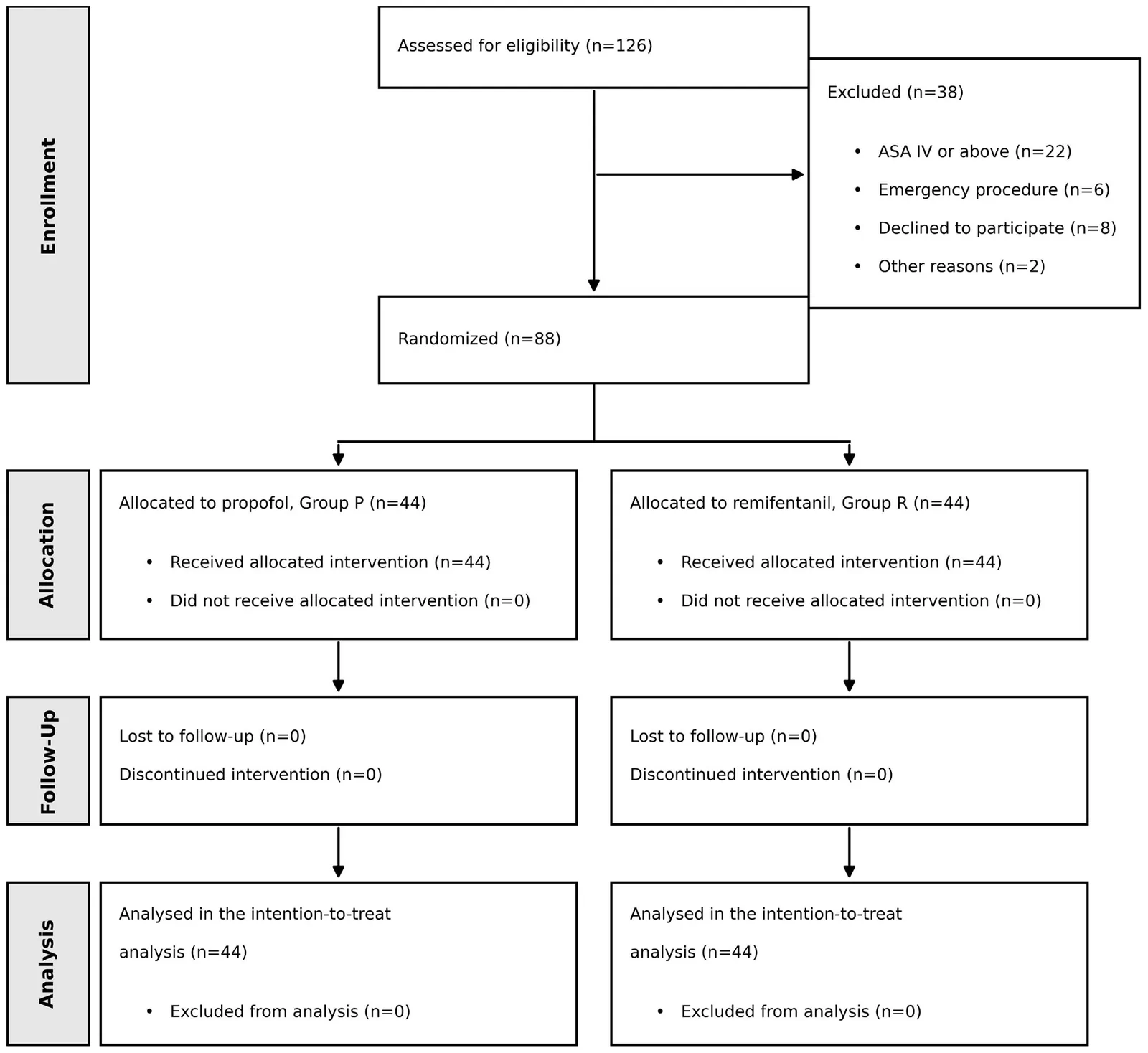
Consolidated Standards of Reporting Trials 2010 flow diagram illustrating participant screening, enrollment, allocation, and analysis. ASA: American Society of Anesthesiologists; ITT: intention-to-treat.

**Table 1.** Demographic characteristics and baseline variables.

Variable	Group P (n=44)	Group R (n=44)
Age, years—median (IQR)	51.5 (43.0–56.0)	52.0 (43.0–56.0)
BMI, kg/m^2^—median (IQR)	25.0 (24–28)	26.0 (26–32)
Sex—n (%)		
Female	28 (63.6%)	25 (56.8%)
Male	16 (36.4%)	19 (43.2%)
ASA score—n (%)		
ASA I	8 (18.2%)	6 (13.6%)
ASA II	28 (63.6%)	26 (59.1%)
ASA III	8 (18.2%)	12 (27.3%)
Total cumulative drug dose—median [IQR]	160 mg (150–170)	200 μg (200–280)

Data are presented as median (IQR) or n (%), as appropriate. Drug dose represents the total cumulative dose administered during the procedure. ASA: American Society of Anesthesiologists; BMI: body mass index; IQR: interquartile range.

### Primary outcome: quality of recovery

BIS values were maintained within the target range (60–80), and RSS scores remained 2–3 throughout. No significant difference was observed in QoR-15 scores (p=0.95): median 140 (136–142) in Group P and 140 (134–144) in Group R, with a median difference of 0 points (95%CI -1.0 to 1.0), well below the MCID of 8.0 points (Table 2).

**Table 2.** Perioperative outcome measures.

Outcome measure	Group P (n=44)	Group R (n=44)	p	RD or MD (95%CI)
QoR-15 score—median (IQR)	140 (136–142)	140 (134–144)	0.946	MD 0 (95%CI -1 to 1)
Procedure duration, min—median (IQR)	8 (8–10)	7 (6–8)	0.016*	–
Time to procedure initiation, sec—median (IQR)	60 (60–120)	47.5 (30–60)	0.016*	–
Awakening time, s—median [IQR]	60 (60–120)	3 (3–10)	<0.001	–
Bradycardia—n (%)	0 (0%)	12 (27.3%)	<0.001	RD -27.3% (-40.4% to -14.1%)
Desaturation—n (%)	0 (0%)	6 (13.6%)	0.026*	RD -13.6% (-23.8% to -3.5%)
PONV—n (%)	0 (0%)	9 (20.5%)	0.003*	RD -20.5% (-32.4% to -8.5%)
Hypotension—n (%)	0 (0%)	0 (0%)	–	–
**Total complications—n (%)**	**0 (0%)**	**27 (61.4%)**	**<0.001**	**RD -61.4% (-75.8% to -47.0%)**
Endoscopist satisfaction: Excellent (5)—n (%)	40 (90.9%)	25 (56.8%)	0.003	RD 34.1% (17.2–51.0%); RR 1.60 (1.22–2.10)
Endoscopist satisfaction: Good (4)—n (%)	4 (9.1%)	13 (29.5%)	–	–
Endoscopist satisfaction: Acceptable (3)—n (%)	0 (0%)	3 (6.8%)	–	–
Endoscopist satisfaction: Poor (2)—n (%)	0 (0%)	3 (6.8%)	–	–

CI: confidence interval; IQR: interquartile range; MD: median difference; PONV: postoperative nausea and vomiting; QoR-15: Quality of Recovery-15; RD: risk difference; RR: risk ratio. Mann-Whitney U test, chi-square test, or Fisher’s exact test applied as appropriate. *p<0.05. Total complications indicates the number of patients who experienced at least one complication.

### Perioperative complications and secondary outcomes

Desaturation occurred exclusively in Group R (13.6%, p=0.026). Bradycardia was recorded in 27.2% of Group R versus 0% in Group P (p<0.001). Total complication incidence was 0% in Group P versus 61.4% in Group R (p<0.001). PONV occurred in 20.5% of Group R patients versus 0% in Group P (p=0.003). Median awakening time was shorter in Group R (3 vs. 60 s; p<0.001). Procedure duration was longer in Group P (8 vs. 7 min; p=0.016). No significant difference in time to discharge was observed. Endoscopist satisfaction was significantly higher in Group P (90.9 vs. 56.8% excellent scores; p=0.003) (Table 2).

## DISCUSSION

Both groups achieved median QoR-15 scores of 140, above the good recovery threshold of 122 points, with a between-group difference of 0 points (95%CI -1.0 to 1.0)—well below the MCID of 8.0 points^9,10^. The absence of a primary outcome difference is the principal finding; secondary safety differences are exploratory and should be interpreted accordingly. Previous comparative studies of sedative agents for gastrointestinal endoscopy have primarily focused on procedural success, cardiopulmonary adverse events, or patient satisfaction rather than patient-reported recovery quality. By using the QoR-15 as the primary endpoint, our study adds a multidimensional patient-centered assessment of early postoperative recovery. These findings suggest that comparable early recovery quality can be achieved despite substantial differences in perioperative safety profiles.

The complication profile in the remifentanil group reflects established pharmacology: μ-opioid receptor activation predisposes to bradycardia, while dose-dependent respiratory depression may precipitate desaturation^5^. All complications were brief and self-limiting, consistent with remifentanil’s rapid offset kinetics. Nevertheless, a total complication incidence of 61.4% represents a relevant safety concern in outpatient practice. The 20.5% PONV incidence is consistent with the emetogenic properties of opioids^13^, while propofol’s antiemetic effects via dopaminergic inhibition explain the complete absence of PONV in Group P. Our findings are consistent with previous studies reporting a higher incidence of opioid-related adverse events during endoscopic sedation with remifentanil, particularly bradycardia and PONV^14,15 16^. In contrast, propofol has consistently demonstrated a favorable safety profile and intrinsic antiemetic properties, supporting its widespread use for ambulatory gastrointestinal endoscopy^17^.

The shorter awakening time with remifentanil (3 vs. 60 s; p<0.001) reflects its context-sensitive pharmacokinetics^5^ but did not translate into reduced time to discharge, indicating that awakening speed alone does not determine discharge readiness. Rather, discharge after ambulatory gastroscopy depends on overall clinical recovery, including hemodynamic stability, adequate oxygenation, and the absence of treatment-related adverse events. Higher endoscopist satisfaction with propofol is consistent with the broader literature^17,18,19,20^.

The present study has several strengths. Its prospective randomized controlled design minimized selection bias, while sedation depth was standardized using both BIS monitoring and the Ramsay Sedation Scale to ensure comparable sedation targets between groups. In addition, the QoR-15 questionnaire provided a validated patient-centered assessment of recovery, and complete follow-up of all randomized participants minimized the risk of attrition bias.

Limitations of this study include its single-center design and the inability to achieve complete endoscopist blinding. In addition, the QoR-15 questionnaire was administered 30 min after the procedure to assess early postoperative recovery before discharge because all participants underwent ambulatory day-case gastroscopy. Consequently, our findings reflect immediate recovery rather than the conventionally validated 24-h recovery profile. Finally, a ceiling effect was observed in both groups, which may have reduced the sensitivity of the QoR-15 to detect subtle differences in recovery quality.

## CONCLUSION

Propofol and remifentanil provided comparable patient-reported recovery quality in elective gastroscopy. The 61.4% total complication incidence with remifentanil—versus zero with propofol—and the absence of a discharge time benefit support propofol as the preferred agent in routine outpatient gastroscopy. Remifentanil may remain appropriate where propofol is contraindicated, or rapid offset is specifically required. Larger multicenter trials are warranted.

## Data Availability

The datasets generated and/or analyzed during the current study are available from the corresponding author upon reasonable request.
